# Heart Failure: The Need for Global Health Perspective

**DOI:** 10.2174/1573403X11309020001

**Published:** 2013-05

**Authors:** Amitava Banerjee, Shanthi Mendis

**Affiliations:** 1 University of Birmingham Centre for Cardiovascular Sciences, City Hospital, Birmingham B18 7QH, UK;; 2 Management of Noncommunicable Diseases, Noncommunicable Diseases and Mental Health Cluster, World Health Organization, Geneva, Switzerland

**Keywords:** Heart failure, epidemiology, valve disease, treatment, prognosis, prevention.

The global burden of cardiovascular disease (CVD), whether in terms of mortality or morbidity, is not in question and the scale of the threat to global health and security has been well documented and highlighted by both the scientific literature and last year’s United Nations High-Level Meeting [1-5]. 

Heart failure (HF) is an important cardiovascular disease in its own right, a risk factor for disease (atrial fibrillation, stroke, coronary artery disease), and a consequence of other diseases (e.g. rheumatic, hypertensive or coronary artery disease), therefore posing a triple threat to public health [6-12]. Heart failure is an important cause of hospitalisation and incurs huge costs to the individual and to society [4]. HF represents the common final pathway for all forms of cardiac disease, regardless of the aetiology. The role of HF in all stages in the development of CVD makes it an important focus for prevention and control of CVD, and for broader health policy approaches. 

This timely issue examines the burden of heart failure in terms of epidemiology, treatment and trends in policies in different regions of the world. Together, these contemporary reviews present an opportunity to compare and contrast the challenges faced by all areas of the world [13-20]. Although there are geographic differences in aetiology, with Chagas disease in South America, rheumatic heart disease in the Indian subcontinent and nutritional cardiomyopathies in Africa, common problems of inadequate access to proven primary and secondary prevention therapies are ubiquitous [21] particularly in low and middle income countries.

Western countries are over-represented in population-based studies of epidemiology, aetiology, prognosis and management of HF, and in clinical trials, whereas the regions of the world carrying the greatest burden are under-represented and prospective studies are rare. While advances in device therapy and diagnostics are included in consensus practice guidelines in developed countries [22, 23], many countries of the world struggle to offer the inexpensive, essential medicines to prevent and treat HF patients [24]. This lack of access to generic diuretics and beta-blocker therapy is even more striking given the inclusion of HF patients from low-resource settings in trials of novel therapies [25]. However, this series of reviews highlights that in all parts of the world, there is considerable room for improving equity of diagnosis and treatment. 

As well as lack of population-based, prospective data for HF, the existing data are heterogeneous in terms of population (hospital, community and specific patient subpopulations by age, sex or aetiology), definition and outcome (hospitalisation, mortality, prevalence, incidence). These factors make direct comparison of data from different settings challenging. Clear consensus guidelines exist for definition and diagnosis of HF [22-23] but should be adhered to in future epidemiologic studies, particularly from low- and middle-income settings in order to give an accurate picture of the global burden of HF and in order to improve comparability. Estimates of incidence rates in South Asia, East Asia, Africa and the Middle East are likely to under-estimate true burden of disease due to these methodological issues. Where burden of HF is estimated from data regarding risk factors for HF, such as coronary artery disease and hypertension [26], consistency in the definitions of these risk factors is also required in order to improve accuracy and comparability of data [27]. Encouragingly, there are increasing numbers of registries which aim to better quantify burdens of rheumatic heart disease, coronary artery disease and atrial fibrillation, particularly in low- and middle-income settings [8, 28, 29]. However maintaining the data quality of these registries in resource constrained settings remains a major challenge.

HF is likely to grow as a major clinical and public health challenge due to demographic changes as well as rising prevalence of causative risk factors in aging patients, including hypertension, coronary artery disease, degenerative valve disease and obesity. Early identification of patients at risk for HF and asymptomatic patients with structural heart disease through a primary health care approach is critical if the human morbidity and mortality toll and the economic burden that HF causes are to be decreased. Improved management of individuals with HF is a key component of primary prevention of stroke, atrial fibrillation and other important causes of CVD [30], and is recognized as an essential intervention even for resource constrained primary care settings [31]. The need for intersectoral and integrated approaches has been repeatedlystressed by successive global health consensus documents [2, 3, 12, 32]. Heart failure has earned its place as part of these intersectoral approaches (across cardiac diseases, other disease areas and other disciplines), which should be global in their scope and the need is urgent.

In 2011 the World Health Assembly adopted a global target of 25% reduction of premature mortality from major noncommunicable diseases including cardiovascular disease by 2025. Strengthening primary health care worldwide, for primary and secondary prevention of HF will be critical for achieving this ambitious global target. 
